# The Central Role of the NAD+ Molecule in the Development of Aging and the Prevention of Chronic Age-Related Diseases: Strategies for NAD+ Modulation

**DOI:** 10.3390/ijms24032959

**Published:** 2023-02-03

**Authors:** Borut Poljšak, Vito Kovač, Stjepan Špalj, Irina Milisav

**Affiliations:** 1 Laboratory of Oxidative Stress Research, Faculty of Health Sciences, University of Ljubljana, 1000 Ljubljana, Slovenia; 2 Department of Orthodontics, Faculty of Dental Medicine, University of Rijeka, 51000 Rijeka, Croatia; 3 Institute of Pathophysiology, Faculty of Medicine, University of Ljubljana, 1000 Ljubljana, Slovenia

**Keywords:** NAD+ precursors, NAD+ levels, sirtuin, PARP

## Abstract

The molecule NAD+ is a coenzyme for enzymes catalyzing cellular redox reactions in several metabolic pathways, encompassing glycolysis, TCA cycle, and oxidative phosphorylation, and is a substrate for NAD+-dependent enzymes. In addition to a hydride and electron transfer in redox reactions, NAD+ is a substrate for sirtuins and poly(adenosine diphosphate–ribose) polymerases and even moderate decreases in its cellular concentrations modify signaling of NAD+-consuming enzymes. Age-related reduction in cellular NAD+ concentrations results in metabolic and aging-associated disorders, while the consequences of increased NAD+ production or decreased degradation seem beneficial. This article reviews the NAD+ molecule in the development of aging and the prevention of chronic age-related diseases and discusses the strategies of NAD+ modulation for healthy aging and longevity.

## 1. Introduction

Nicotinamide adenine dinucleotide (NAD+) is composed of an adenosine 5′-phosphate coupled to ribosylnicotinamide 5′-phosphate by the pyrophosphate linkage. NAD+ is necessary for more than 500 enzymatic reactions [1] and is of importance in a large number of evolutionarily conserved signaling pathways regulating DNA repair and genomic signaling, apoptosis, senescence, proliferation and endocrine signaling, mainly through NAD+ regulation of sirtuin deacetylases’ (SIRT) activity that affects metabolism, DNA repair, stress resistance, cell survival, inflammation, mitochondrial function, lipid and glucose homeostasis by targeting transcription factors (FOXO3a, PGC-1α, p53, NF-κB, HIF-1α), and many other cellular targets [2]. Additionally, NAD+ is significant in regulating gene expression required for oxidative stress response, catabolic metabolism and mitochondrial biogenesis. Furthermore, NAD+ influences epigenetics by modulating the acetylation status of histones and other proteins [3]. For example, NAD+ degradation in the aging process increases the consumption of S-adenosylmethionine (SAM) [4], an essential cellular methyl donor. Excessive supplementation with nicotinamide (NAM), nicotinamide riboside (NR) and nicotinamide mononucleotide (NMN) can potentially exacerbate the problem of methylation by increasing NAM levels [5,6,7], as NAM can be methylated to N^1^-methylnicotinamide (MNAM), which lowers methyl donor levels and increases the risk of vascular disease, neurodegenerative disease and chronic kidney disease by producing more homocysteine [8,9]. Unexpectedly, in the long-term study of nicotinamide riboside chloride (Niagen) [10], no increase in homocysteine was observed in subjects who consumed up to 1000 mg of Niagen.

NAD+’s role in electron transfer throughout oxidation–reduction (redox) reactions is essential for ATP production [11]. Furthermore, as a cosubstrate of enzymes, it is cleaved as a result of increased oxidative stress, senescence, poly-(adenosine diphosphate-ribose) polymerases (PARP) activation by DNA damage and NADases activation during inflammation. Despite specific NAD+ subcellular requirements and the difference in NAD+ levels in various cell parts [12], the NAD+ synthesis in general decreases and NAD+ degradation increases during aging, leading to an overall reduction in the concentration of intracellular NAD+ [13,14,15,16,17,18,19,20,21].

## 2. NAD+ Usage and Degradation

NAD+ acts as a coenzyme in the production of energy (glycolysis, mitochondrial respiration) and its reduced form (NADH) is used as a means of energy transfer. During redox reactions, NAD+ is converted from reduced (NADH) to oxidized form (NAD+) but is not degraded [22]. Therefore, these reactions do not change the overall amount of NAD+ in cells. NAD+ is degraded as a rate-limiting substrate for many signaling enzymes, including the sirtuins, SIRT1 and SIRT3, the poly(ADP-ribose) polymerase (PARP) proteins, PARP1 and PARP2, a COOH-terminal binding protein (CtBP), the cyclic ADP-ribose (ADPR) synthetases CD38 and CD157, and various other NAD+-dependent enzymes. PARPs, sirtuins, CD157 and SARM1 degrade NAD+ to NAM and ADP-ribose (ADPR) and due to the difference in affinity for NAD+ (Michaelis–Menten constant, *K*_m_) for NAD+, they have different potential for degradation of NAD+ (for details, see reference [23]). Briefly, the *K*_m_ of SIRT1 ranges around 94 to 888 μM, SIRT2, SIRT4 and SIRT6 may have a *K*_m_ for NAD+ below the physiological range, implying that NAD+ is not necessarily the rate-limiting factor of their activity [24,25,26,27,28]. PARP-1 has *K*_m_ for NAD+ in the range of 20–97 μM [25,28,29,30,31]. Similarly, CD38 and SARM1 have *K*_m_ for NAD+ around 15–25 μM [26,32]. Because the *K*_m_ of PARP1 and CD38 for NAD+ are lower than those of the SIRT1, their activation may limit SIRT1 activation by decreasing the NAD+ content, and inhibition of PARP1 and CD38 can increase the overall availability of NAD+, leading to SIRT1 activation [33]. For example, CD38-deficient mice had 30-fold higher NAD+ levels in their tissues than the wild-type mice [34]. Intracellular NAD+ concentrations are roughly between 0.2 and 0.5 mM; however, these concentrations also differ between the tissue/cell types and cell compartments [28]. The activity of NAD+-consuming enzymes is conditioned by DNA damage (PARPs) and inflammation (CD38, CD157) and is a major contributor to the need to constantly re-synthesize NAD+ [35,36,37]. The NAD+-degrading enzymes CD38, PARPs and sirtuins cause NAD+ depletion in mammalian tissues [21]. NAD+ role in redox reactions affects cellular energy levels and mitochondrial function. NAD+ is an important factor in aging and the development of age-related diseases in the role of a cofactor in redox reactions and a coenzyme in metabolic processes. It also has a decisive role in non-redox reactions, such as cellular signal transduction, when acting as a substrate for sirtuins and PARPs [38]. Sirtuins, PARPs and CD38 all compete for NAD+ in the antagonistic relationship and play different roles regarding aging and life span. Over-activation of PARPs and CD38 negatively influences life span [21,39], while increased sirtuin activation beneficially regulates the organismal life span in several animal models [40,41,42]. Although the exact causes of NAD+ decline with advanced age are not fully understood [21,38], it has been established that NAD+ decline is caused by increased PARP activity due to increased DNA damage. Consequently, the latter lowers cellular NAD+ concentrations up to 80% [43], while a defect in NAMPT-mediated NAD+ biosynthesis due to low-grade chronic inflammation may contribute to this decrease even further [44]. Inflammaging (age-associated inflammation) caused by the cessation of cell division augments NAD+ consumption by activating CD38+ pro-inflammatory macrophages [45]. NAD+ is also phosphorylated by NAD+ kinase to generate NADP+, which in turn is reduced by dehydrogenases to generate NADPH [12].

## 3. Consequences of Decreased NAD+ Levels

Decreased NAD+ levels manifest at organismic, tissue, cellular and mitochondrial levels. Reduced NAD+ alters mitochondrial activity by elevated NADH/NAD+ ratio related to the increased ROS production [46], decreased oxidative metabolism and mitochondrial biogenesis. Such time-dependent progressive increase in mitochondrial dysfunction and collapse of oxidative phosphorylation (OXPHOS) stimulates a shift in metabolism from using mitochondrially produced ATP to dependence on glycolysis as a consequence of the accumulation of hypoxia-inducible factor 1 alpha (HIF-1α) and decreased activity of respiratory complexes I, III and IV [47], which stimulates the Warburg effect involved in the metabolic syndrome, obesity, type 2 diabetes, the onset of cancer and other degenerative diseases [47,48,49,50,51]. High concentrations of accumulated intracellular NADH may furthermore impede OXPHOS by promoting pyruvate to lactate conversion and permeability reduction of the voltage-gated anion channel in the outer mitochondrial membrane [52]. Additionally, obesity triggers the vicious cycle by decreasing the enzyme nicotinamide phosphoribosyl transferase (NAMPT), which catalyzes the rate-determining step for NAD+ synthesis and NAD+ levels in human tissues. A decrease in NAMPT-mediated NAD+ biosynthesis in adipocytes seriously compromises insulin sensitivity in multiple organs [53]. A reduction in systemic NAD+ biosynthesis also decreases NAD+-dependent deacetylases (sirtuin) activity, which regulates glucose-stimulated insulin secretion [54]. The elevated glucose levels, impaired insulin secretion and insulin resistance interconnected with the development of advanced glycation end products (AGEs) are characteristics of type 2 diabetes [55] and are a contributing determinant of the pathogenesis of cardiovascular diseases (CDs) [56,57]. The mice with a shortage of SIRT3 on a high-fat diet develop symptoms similar to metabolic syndrome in humans, leading to accelerated obesity, insulin resistance, hyperlipidemia, steatohepatitis and chronic inflammation [58]. On the other hand, when the NAD+ level was elevated with NAD+ booster niacin for a period of 4 to 9 months, blood and muscle NAD+ level, OXPHOS function, mitochondrial mass and biogenesis, muscle mass and strength increased, and disease symptoms improved in patients with mitochondrial myopathy, while the percentage of whole-body fat and hepatic/visceral fat decreased by 25 and 50%, respectively, although subcutaneous adipose tissue remained unchanged. In addition, niacin therapy increased HDL, reduced apolipoprotein B particles and did not affect total cholesterol in the treated patient group [59]. Furthermore, increased levels of NAD+ by genetic manipulation (e.g., CD38^−/−^ or PARP1^−/−^) protected animals from weight gain [60].

NAD+ half-life ranges from 1 to 2 hours in the cytoplasm and nucleus and about 8 hours in the mitochondria [61,62]. According to Zhu and coworkers [63], 3 g of nicotinamide released from NAD+ consumption is necessary for resynthesizing to NAD+ a few times daily in a 75 kg person to achieve NAD+ homeostasis. Intracellular NAD+ concentrations have the range 100–120 µM in the nucleus and 50–100 µM in the cytoplasm [62,64,65]. The total NAD+ levels in mammalian cells appear to be between 200 and 500 μM; higher levels of NAD+ are needed in metabolically active cells like neurons and cardiac myocytes [46]. Intracellular NAD+ concentrations vary between cell compartments, cell types, cellular states, and growth conditions [65].

**Figure 1:**
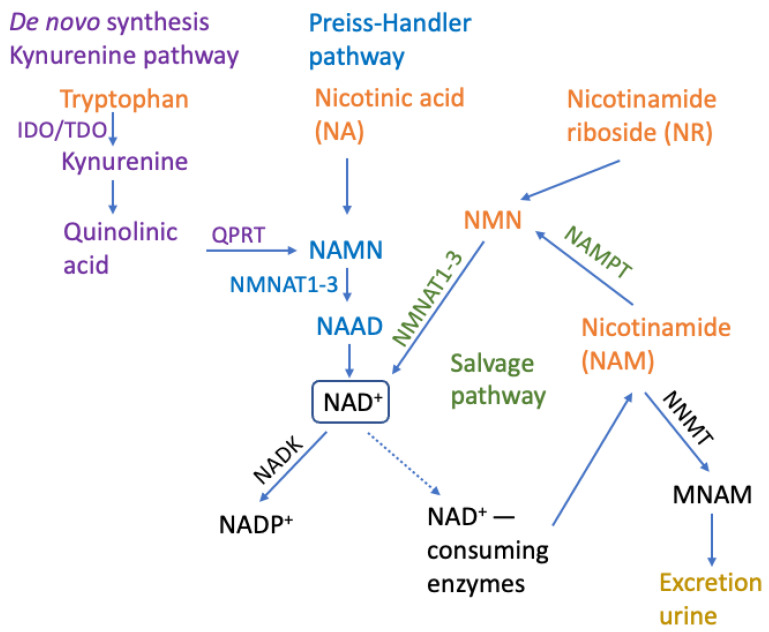
NAD+ metabolism. The two biosynthetic pathways, Kynurenine/De novo synthesis and Preiss–Handler pathway, with crucial steps and enzymes are indicated in violet and blue, respectively, and the salvage pathway is in green. The common NAD+ precursors available from diet are in orange. NAD+ consumption and degradation pathways and enzymes are in black. NAD+-consuming enzymes are PARP1-17, SIRT1-7 and glycohydrases/NADases: CD38, CD157 and SARM1. IDO: indoleamine 2,3-dioxygenase; TDO: tryptophan 2,3-dioxygenase; QPRT: quinolinate phosphoribosyltransferase; NAMN: nicotinamide mononucleotide; NMNAT1-3: nicotinamide mononucleotide adenylyltransferases; NAAD: nicotinic acid adenine dinucleotide; NAD+: nicotinamide adenine dinucleotide; NADK: NAD+ kinase; NADP+: nicotinamide adenine dinucleotide phosphate; NNMT: nicotinamide N-methyltransferase; MNAM: N^1^-methylnicotinamide; NAMPT: nicotinamide phosphoribosyltransferase; NMN: nicotinamide mononucleotide.

NAD+ may be maintained, restored and re-used via three independent biosynthetic pathways: (1) De Novo Synthesis (from L-tryptophan through the kinurenine pathway), (2) Preiss–Handler Pathway (from nicotinic acid or nicotinic acid ribose to generate NAMN, which is then transformed into NAAD and NAD+) and (3) Salvage Pathway (from niacinamide/nicotinamide, which is generated as a by-product of the enzymatic activities of NAD+-consuming enzymes) (Figure 1) [28,42,66,67]. Nicotinamide salvage pathway, where NAD+ is synthesized from dietary precursors NR, NAM and nicotinic acid (NA), is a dominant pathway enabling stable cellular concentrations of NAD+ in the majority of tissues. The NAMPT functions as the rate-limiting enzyme of the aforementioned pathway [22] generating NMN and pyrophosphate (PP) from NAM and α-D-5-phosphoribosyl-1-pyrophosphate (PRPP). Adenylyltransferases (NMNAT1-3) then form NAD+ from NMN and ATP [68].

## 4. Methods to Increase NAD+ Levels

NAD+ levels are reduced during aging due to increased oxidative stress and chronic inflammation, which dysregulate NAD+ metabolism by activating CD38 and PARPs or inhibiting NAMPT [17,69]. The prevention of NAD+ degradation and the increased availability of NAD+ can influence, delay and even somewhat reverse the aging process and age-related diseases [13,14,15,16].

Many studies have documented the genetic or pharmacologic restoration of NAD+ in mice to enhance longevity and healthspan [22,27,38,44,47,70,71,72,73,74,75,76,77,78,79]. NAD+ levels can also be regulated by lifestyle and selected nutritional interventions [80]. Accordingly, NAD+ levels may be elevated in mammals by ingesting NAD+ boosters/intermediates, e.g., nicotinamide mononucleotide, nicotinic acid, nicotinamide, tryptophan and nicotinamide riboside [27,75,81,82,83,84,85,86]. They can also be increased by decreasing NAD+ utilization by PARP enzymes or CD38/CD157 with the use of PARP, CD38 and SAM1 inhibitors [43,83,87,88,89,90,91].

By application of NAD+-replacement therapy with NAD+ precursors NR and NMN supplementation, higher concentrations of NAD+ were observed in mice and humans [27,44,92]. However, it has been reported that CD38 is the central enzyme causing the degradation of the NAD+ precursor nicotinamide mononucleotide (NMN), [21] which can prevent the generation and increase in NAD+ levels from supplementation with NAD+ boosters. Aging is characterized by NAD+ degradation via CD38 as its expression and activity increase with age. Knockout of CD38 inhibits age-related NAD+ degradation, activates sirtuins and mitochondrial function, and prevents age-related metabolic disorders [21]. This may explain why mere supplementation with precursors is insufficient to raise NAD+ levels. There are no observed effects of NR supplementation on the mitochondrial respiration, content or morphology reported in skeletal muscles of pathologically overweight and insulin-resistant males [93]. Similarly, it was reported that daily supplementation with 1000 mg NR for 6 wk did not raise NAD+ levels of skeletal muscles in healthy overweight or obese persons of both genders [94]. NR effectively stimulates NAD+ metabolism and elevates NAD+ [95] in normal-weight disease-free middle-aged and older adults, indicating the potential link between obesity and CD38. One such link could represent circulating lipopolysaccharides (LPS) released by gram-negative bacteria, which are associated with infection and inflammation and are elevated in persons with a BMI of 30 compared to a BMI of 24 [96]. Exposure of macrophage cells to LPS results in CD38 increased gene expression [97]. M1-like macrophages that tend to cause inflammation in visceral white adipose tissue build-ups during aging have significant levels of the NAD+-consuming enzyme CD38. In addition, senescent cells accumulate in visceral adipose tissue during aging and inflammatory cytokines secreted by senescent cells incite macrophages to proliferate and express CD38 [45]. Obesity, LPS and CD38 all increase during aging [17,98,99]. Although NR is resistant to CD38 enzymatic activity in vitro, CD38 can decrease NAD+ levels in vivo due to NR supplementation, as NR is converted to NMN [100,101]. Namely, NMN is either derived from NAM by NAMPT or NR by NR kinase [12].

It can be concluded that the prevention of aging and metabolic disorders can be strongly impacted with the NMN and NR therapies by decreasing LPS, inflammation and CD38 levels with CD38 inhibitors such as flavonoids: epigenin, luteolin, quercetin, kuromanin and thiazoloquin(az)olinones, like the compound **78c** [102,103,104,105]; PARP pharmacological inhibitors have also been reported to increase NAD+ levels [60,106]. It is also possible to increase NAD+ levels by activating NAD+-generating enzymes, the most important of which is NAMPT, which converts NAM to NMN, thereby increasing the production of nicotinamide mononucleotide (NMN), which is the major NAD+ precursor in mammalian cells in the salvage pathway (Figure 1) [107]. NAMPT biosynthesis can be modulated by the small synthetic molecule activators P73C and SBI-797812 [107] or a natural compound notoginseng leaf triterpenes and a natural peptide IRW (Ile-Arg-Trp), which also activate *Nampt* gene expression and increase intracellular NAMPT protein abundance [108,109,110]. Important and well-researched lifestyle approaches to extending lifespan include aerobic exercise, fasting, glucose deprivation and caloric restriction, which increase NAD+ [2,89,90] by activating the enzyme NAMPT, the rate-limiting NAD+ biosynthetic enzyme in mammals [111,112,113,114]. Another approach to affect NAD+ levels can be achieved by inhibiting the enzyme nicotinamide N-methyltransferase (NNMT), which converts nicotinamide to methylnicotinamide, to increase nicotinamide levels, which can then be converted to NAD+ [28,115,116,117,118,119,120,121,122]. Both NR and NMN can increase nicotinamide levels, thereby enhancing NNMT activity due to higher substrate availability [123]. NNMT activity can affect NAD+ biosynthesis, as well as drive epigenetic modifications and influence gene expression by modulating the intracellular methylation index (SAM/SAH ratio) [124] by catalyzing the N-methylation of nicotinamide, using S-adenosyl-L-methionine (SAM) as a methyl donor, resulting in N^1^-methylnicotinamide (MNAM) and releasing S-adenosyl-L-homocysteine (SAH) [124].

Pharmacological inhibition of CD38, PARPs and NNMT and NAMPT activation can modify NAD+ levels, yet not enough is known about the safety in human (non-oncological) treatments (e.g., in the prevention of aging and amelioration of chronic diseases). Additionally, caloric restriction upregulates SIRT1 in adipocytes and promotes lipolysis and free fatty acid mobilization by suppressing the nuclear hormone receptor PPARγ, which promotes adipogenesis [125]. Aerobic sporting activity adds to the total accumulation of NAD+ induced by the skeletal muscle NAMPT expression [126]. These activities also stimulate the NAD+ salvage pathway through the 5′-AMP-activated protein kinase (AMPK) [111] and thus modify the age-dependent decrease in NAD+ [127]. The application of the exercise mimetics, such as 5-aminoimidazole-4-carboxamide-1-β-D-riboside, resulted in AMPK-induced increase in NAD+, increased sirtuin activity, oxidative mitochondrial activity and improved endurance [85]. Therefore, the optimal approach to increasing NAD+ tissue levels integrates the NAD+ precursors, NAD+ degradation prevention and exercise/caloric restriction.

## 5. Beneficial Effects of NAD+ Boosting

**Figure 2:**
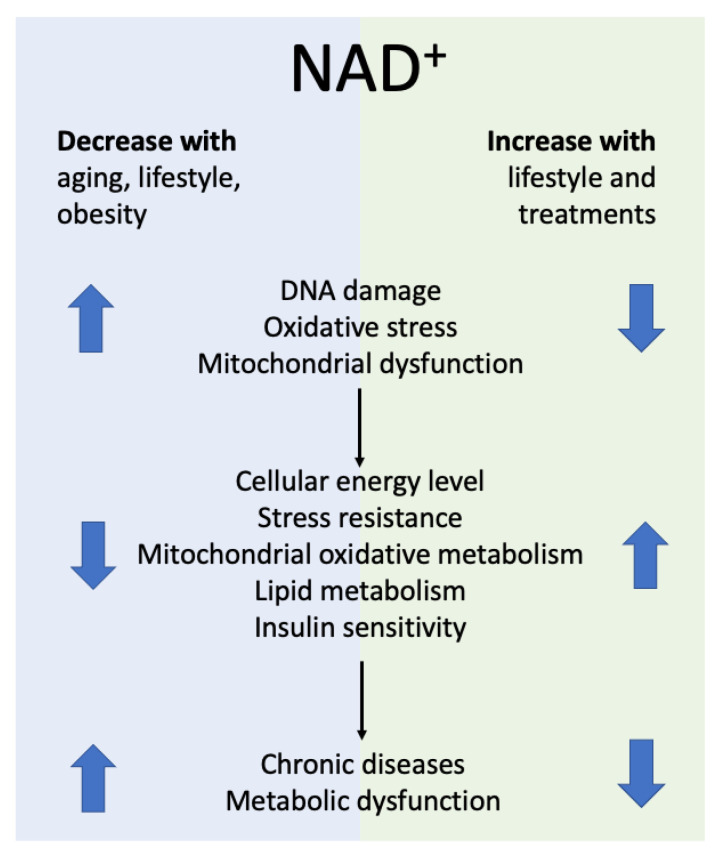
Association of NAD+ bioavailability and healthspan.

Increasing NAD+ levels restores mitochondrial function and ameliorates mitochondrial disorders [47,75,128,129] and the age-associated disorders observed in various mouse models of human disease (Figure 2). Scientific literature reports that rising NAD+ levels influence several different conditions and illnesses, such as metabolic syndrome, type 2 diabetes and/or insulin sensitivity [27,53,71,86], cancer [2,51,130,131,132], cardiovascular disease [75,133,134,135,136,137], neurodegeneration [138,139,140], renal function [141,142] and Alzheimer’s disease [77,143]. It also reduces inflammation [47,144,145], helps prevent obesity [27,53,146,147,148] and influences life extension [71,144,149,150].

Clinical trials with small participant numbers indicate that NAD+ boosters could elevate NAD+ levels in volunteers and are relatively safe for humans [59,81,95,151,152,153]. For example, orally administered NR increased NAD+ 2.7-fold in human blood in 12 subjects with only one dose of 1000 mg applied per os [81]. Similarly, Airhart and coworkers [152] documented that the NAD+ amount was doubled by oral ingestion of NR (250–1000 mg/d) after nine days in 140 healthy volunteers. In the recent study by Fahy et al. [154] the human epigenetic clock, predicting their biological age, was for the first time turned back by 2.5 years with a combination of growth hormone, metformin and dehydroepiandrosterone, through the thymus and immune system regeneration and a decrease in the CD38 enzyme activity, which conserves NAD+.

## 6. The Effects of NAD+ on Cancer and Inflammation

The increase in cancer risk due to impaired genome stability is related to NAD+ depletion in aging because NAD+ levels regulate cellular energy production, cellular DNA repair, signal transduction and genome stability. This topic has been presented in detail in [2]. In short, sufficient NAD+ levels could prevent or reverse the phenotype of malignant cells at early stages by inducing cellular repair and adaptive response to stress and regulating cell cycle arrest and apoptotic clearance of damaged cells. Thus, adequate amounts of NAD+ have a protective role in genomic stability, mutation formation and cancer prevention. In contrast, during cancer promotion, progression and treatment, elevated NAD+ levels could have deleterious effects on the malignant process due to growth benefits, increased resistance and prolonged cell survival as NAD+ deficiency regulates oncogene-induced DNA damage and tumor development [155].

Sirtuins are involved in carcinogenesis and cancer prevention as they regulate genes involved in the process of DNA repair and maintenance [156]. For example, the activity of mammalian SIRT1 depends on the NAD+/NADH ratio. By increasing cellular NAD+ levels, AMPK increases SIRT1 activity, leading to deacetylation and modulation of the activity of downstream SIRT1 targets. In contrast, decreased levels of NAD+ reduce the efficacy of sirtuins (SIRT1) that deacetylate tumor suppressor proteins such as p53 [157], which are involved in cell cycle arrest, apoptosis and autophagy [156,158]. It has been observed that low NAM levels acting as NAD+ precursors are beneficial for SIRT1 activity, whereas the opposite, NAM accumulation, could be detrimental by inhibiting SIRT1 [159,160,161].

The tumor suppression ability has also been reported for SIRT2 and SIRT3. SIRT3 acts as a tumor suppressor by suppressing ROS through the activation of antioxidant defense by the manganese superoxide dismutase (MnSOD), an essential mitochondrial antioxidant enzyme, and regulating HIF-1 [162,163,164,165]. SIRT2 acts as a tumor suppressor by preventing chromosomal instability during mitosis [166], by regulating the microtubule network [167], by increasing FOXO DNA binding and enhancing the expression of FOXO target genes [168], and preventing oxidative stress-induced death [169] by increasing the expression of antioxidant enzymes such as MnSOD, glutathione peroxidase and catalase [170]. The involvement of NAD+ in apoptosis remains controversial [171], as it could be both pro- and antitumorogenic. Although sirtuins play an important role in tumorigenesis, their role in various aspects of the carcinogenesis process remains debatable. For example, SIRT1 has been observed to both promote and suppress tumor growth [172]. It appears that sirtuins may have a cell-protective function during stress, preventing cells from developing damage. In contrast, they could prevent apoptotic death, stimulate proliferation and facilitate acquired resistance, thus promoting cancer stem cell survival [173]. Sirtuins could give cancer cells a growth advantage [174] by preventing cell loss through apoptosis and senescence-like growth arrest. Elevated NAD+ levels could also increase resistance to radio- and chemotherapy, promote inflammation [2,175] and stimulate angiogenesis [176].

Elevated NAD+ levels may also play an opposing role at different stages of sepsis. For example, activation of SIRT1 has been reported to have a beneficial effect in the initial (proinflammatory) phase [177], whereas SIRT1 expression should be reduced in the later stages of sepsis [178]. NAD+-mediated suppression of AMPK kinase leads to suppression of p53-mediated inhibition of p38 mitogen-activated protein kinase (MAPK) and enhanced NF-κB activity. It affects inflammatory signaling of senescent cells in vivo through higher mobility group A (HMGA) proteins and NAMPT expression, which promotes the proinflammatory senescence-associated secretory phenotype (SASP) [179], thereby exacerbating SASP-associated inflammation [180].

Caution should be exercised in the use of NAD+ and its precursors if aging is a defense mechanism against cancer. Despite these concerns, no evidence has been found that treatment with NR or NMN for a prolonged period of time stimulates tumor development in animals [71,75].

## 7. Side Effects of NAD+ Precursors Observed in Human Trials

Although several small human clinical trials have been conducted and the results imply that increased NAD+ levels by NAD+ boosters are safe in humans (reviewed in [181]), long-term safety studies are lacking to determine the proper dose of NAD+ boosters and treatment duration for aging prevention and as chronic disease therapy. During administration of NAM, NR and NMN, few minor and relatively infrequent side effects were reported in clinical studies, including diarrhea, nausea, rashes, skin flushing, calf cramps, thrombocytopenia, erythema pruritis, skin burning, fatigue, abdominal discomfort and headache [182,183]. NA in high doses can cause hot flashes and elevated blood glucose as well as elevated homocysteine levels.

Nicotinamide (NAM) overdose can cause hepatotoxicity in rare cases [184]. NA and NR decreased physical performance in young rats [185] and the capacity for high-intensity exercise in humans [186]. In contrast, cardiopulmonary performance improved after 6 weeks of NMN supplementation in 48 recreationally trained runners [187], and chronic oral NMN supplementation for 12 weeks significantly improved muscle strength and performance in 10 athletes [188].

## 8. The Future Research Directions and Strategies

Currently, uncertainties remain regarding the pharmacokinetics and pharmacodynamics, tissue specificity of NAD+ boosters and dosing, safety and side effects of chronically elevated NAD+ levels by supplementation with NAD+ precursors or by inhibitors of NAD+-consuming enzymes. Since all NAD+ precursors (NMN, NAM, NR, nicotinic acid and tryptophan) increase the availability of NAD+, the question arises as to which precursor is better and more suitable for human consumption. All of them are naturally present in different foods, thus it may not be a priority to invest money in large human trials. The precursors differ in their potency with respect to increasing NAD+ synthesis and in the number of steps required to form NAD+ in the biochemical pathways. Observed differences also result from the expression of NAD+ biosynthetic enzymes and the preference for specific NAD+ precursors [189]. Subcellular distribution and needs, tissue specificity and efficacy of NAD+ boosters in increasing NAD+ levels in humans should be studied in more detail. To determine which age-related degenerative diseases (e.g., cardiovascular, metabolic, inflammatory, neurodegenerative, muscle-damaging, mitochondrial and cancer) can be ameliorated, prevented or merely delayed with which precursor, further studies using different precursors in the same trial are needed. In addition, to investigate NAD+’s role in promoting health and longevity, studies should also be conducted in healthy populations.

## 9. Conclusions

Although it is difficult to prove causality experimentally, the studies presented support the hypothesis that the decline of NAD+ significantly contributes to aging, chronic diseases and metabolic dysfunction. Metabolic syndrome (e.g., increased blood sugar, decreased insulin sensitivity/insulin resistance), decreased ATP synthesis, reduced mitochondrial function, increased visceral adipose tissue, diabetes, cancer, atherosclerosis, etc., may result also from NAD+ decrease with age. NAD+ is related to aging and the occurrence of age-associated chronic diseases because NAD+-driven processes enable cells and organisms to maintain their high organization, while preserving a lower entropy state [190], which leads to a better health span and life expectancy [113]. NAD+ steady-state levels depend on the ratio between NAD+ synthesis and utilization [12] and can be replenished by three strategies, including (i) reduction of NAD+ degradation by NAD+ consumers (e.g., CD 38 and CD 157) through their inhibitors, such as naturally occurring flavonoids apigenin and luteolinidin [103,113,191], (ii) increasing NAD+ supply through NAD+ precursors (e.g., nicotinamide, niacin, NMN and NR) and (iii) activation of NAD+ generating enzymes, such as NAMPT, as well as converting NAM to NMN in the salvage pathway by NAD+ biosynthesis modulators [107,192].

The extensive research on animals confirmed the interdependence of NAD+ and the organism’s maintenance of full function. Nevertheless, several additional studies on the molecule NAD+ are desirable to elucidate the comprehensive role of NAD+ decay in the causation of aging and human age-associated chronic diseases. Current knowledge of the NAD+ beneficial impact on aging and healthspan is grounded primarily on the research on cell cultures and model organisms and tentatively on the beneficial anti-aging effects of NAD+ in human beings—including increased vitality, reduction in all-cause mortality and an extended healthspan. The latter requires additional corroboration by further extensive research and clinical trials.

There are no long-term safety studies on NAD+ boosters aimed at determining the optimal effective dose, treatment period, bioavailability, metabolism and tissue specificity. The long-term consequences of increased NAD+ levels and the best approaches and combinations to increase the NAD+ levels also need to be elucidated. Further research will answer these crucial questions.

## Data Availability

Not applicable.
